# Galli-Galli Disease: A Rare Acantholytic Variant of Dowling-Degos Disease

**DOI:** 10.1155/2011/703257

**Published:** 2011-05-05

**Authors:** J. Gomes, J. Labareda, I. Viana

**Affiliations:** ^1^Serviço de Dermatologia e Venereologia, Hospital de Braga, Apartado 2242, 4701-965 Braga, Portugal; ^2^Dermatology Department, Centro de Dermatologia Médico-Cirúrgica de Lisboa, Lisboa, Portugal

## Abstract

Galli-Galli disease is a rare acantholytic variant of Dowling-Degos disease, with few cases reported in the literature. We describe a case of Galli-Galli disease and review the literature.

## 1. Introduction

Galli-Galli disease (GGD) is a very rare variant of the Dowling-Degos disease (DDD) with the histologic finding of focal acantholysis. It was originally reported by Bardach et al. in 1982, who described the disease in two brothers and named it eponymically after this family [1].

## 2. Case Report

**Figure 1:**
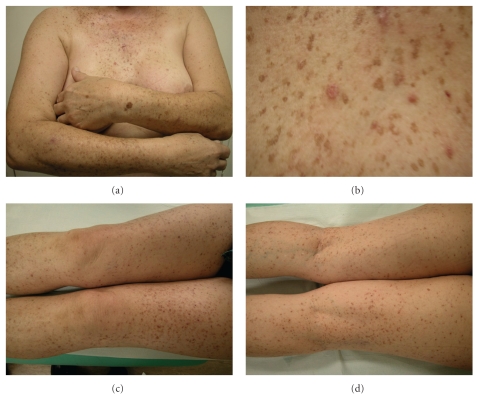
Polymorphous dermatosis characterized by disseminated erythematous papules and plaques and multiple brown macules.

**Figure 2:**
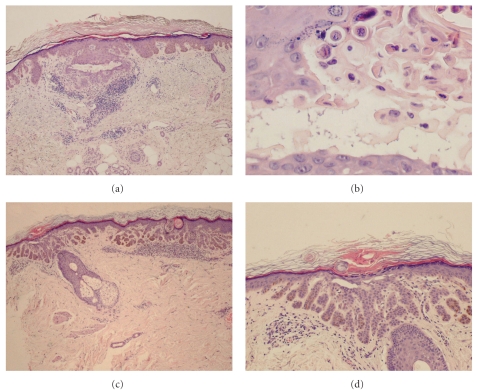
Hematoxylin and eosin. (a) Discrete hyperkeratosis, elongated rete ridges with digitiform projections, and focal acantholysis with formation of linear clefts. Presence of mixed dermal inflammatory infiltrate. (b) Dyskeratotic cells in the interior of a acantholytic cleft. (c) and (d) Digitiform elongation of the rete ridges, with hyperpigmentation of the basal layer confined to the tips of the rete ridges. Suprabasal acantholysis.

A previously healthy 67-year-old Caucasian woman presented with a 15-year history of widespread skin lesions. She had recurrent pruritic papular eruptions and slowly progressing brownish lentigo-like macules. The skin lesions began on the trunk and then became generalized, involving the extensor and flexural surfaces of extremities, including the back of the hands, neck, and trunk. Clinical examination revealed numerous generalized symmetrically distributed brownish macules and some pruritic, hyperkeratotic, erythematous papules (Figure 1). The face, palms, and soles were spared, and she had no nail, hair, teeth, or mucosal alterations. There was no atrophic or hypopigmented lesions. No family history of similar lesions was known. She was previously treated with topical corticosteroids without improvement. Laboratory examinations demonstrated no abnormalities. Histopathologic examination of three biopsy specimens revealed digitiform epithelial downgrowths of the rete ridges, with hyperpigmentation of the basal layer confined to the tips of the rete ridges. Multiple foci of suprabasilar acantholysis were also observed (Figure 2) and a mixed dermal inflammatory infiltrate. In one of the biopsy specimens, we found acantholytic and dyskeratotic cells (Figure 2(b)). Direct immunofluorescence was negative.

Taken together, clinical and pathological data suggested a diagnosis of Galli-Galli disease. Treatment was very difficult with only short-term partial improvement using acitretin (25 mg/day) and topical corticosteroids. 

## 3. Discussion

Galli-Galli disease is a benign but very pruritic and unaesthetic genodermatosis. Its mode of inheritance is believed to be autosomal dominant with variable penetrance, but it can occur sporadically [2, 3], as in our patient. As with DDD it is believed to be linked to mutations in the KRT5 gene [3–6]. Clinically it is characterized by reticulated hyperpigmentation predominantly affecting the flexures along with pruritic, erythematous, scaly papules, similar to the DDD. Histopathologic examination reveals digitiform elongation of the rete ridges seen in DDD, together with suprabasal focal acantholysis [2–9].

A few patients have been described in the literature. A literature search revealed 9 previous case reports of GGD, with a total of 17 patients (Table 1). According to the reported cases, the age range of presentation is 15 to 67 years, 11 patients were male, and 6 were female. All the patients in the reports had reticulated hyperpigmentation in the flexural areas, with the exception of one. In our patient, the characteristic hyperpigmentation of the axillae and inguinal region could not be detected, rendering a diagnosis of GGD difficult.

In 15 of the 17 cases a skin biopsy was performed, and in all of this cases acantholysis was identified, but dyskeratosis was present in only 3 cases. In our case we also identified dyskeratotic cells in one of the fragments, closely resembling features of Darier's or Grover's diseases. However, Darier's disease could be ruled out by the presence of areas of digitate proliferations of the rete ridges, clinically it starts early, lentigo-like macules are rare, and it is commonly associated with nail and mucous membrane involvement that was absent in our patient. In Grover's disease involvement of the distal extremities, as in our patient, and the presence of brown lentigo-like macules are not common. Histologically, areas of lentiginous elongations of the rete ridges are not observed in Grover's disease. 

Acantholysis is a *sine qua non* condition for the diagnosis of Galli-Galli disease [2–9], but dyskeratosis is not an essential finding [2, 3], although its presence does not exclude the diagnosis. 

Because of the overlap of clinical features, to the majority of authors GGD is best considered as an acantholytic variant of Dowling-Degos disease rather than an entity of its own [2, 3]; however, some authors defend that if one accepts the histopathological finding of acantholysis as a pathognomonic feature, GGD should be classified as its own disease entity [8]. 

## Figures and Tables

**Table 1:** Reported cases of Galli-Galli disease.

Author/year	Age	Sex	Description	Acantholysis	Dyskeratosis
Bardach et al.1982 [1]	19	M	Reticulated hyperpigmentation of the ace and neck and pruritic, erythematous papules on flexures, trunk, and extremities	+	+
	15	M		+	+

Mittag et al. [10]	56	M	Hyperkeratotic papules Hyperpigmentation of the folds	+	−
	51	M		+	−
	46	F		No biopsy was performed	
	17	M			

Rutten and Strauss1995 [7]	24	M	Reticulated hyperpigmentation, comedo-like lesions	+	?

De Deene and Schulze 1996 [9]	59	F	Hyperpigmentation of the main folds and trunk, pruritic papules	+	?

Braun-Falco et al. 2001 [2]	53	M	Reticulate hyperpigmentation of the main folds, pruritic papules on the neck, trunk, axilla, and hands	+	−

El Shabrawi-Caelen et al. 2007 [6]	65	F	Recurrent maculopapular eruption involving the trunk and lower extremities	+	+
	67	F		+	−

Sprecher et al. 2007 [5]	43	F	Pruritic papular rash of the flexural areas and reticulate pigmentation	+	−

Gilchrist et al. 2008 [3]	41	M	Hyperpigmentation of the flexures, pruritic papules on the trunk, and proximal extremities	+	−

Müller et al. 2009 [8]	41	M	Erythematous macules and reticulate pigmentation of the main folds, without pruritus	+	−
	52	M		+	−
	48	F		+	−
	25	M	Pigmented macules on the trunk and neck, erythematous, pruritic papules, and perioral scars and comedo-like lesions	+	−
